# ATM/MAPK7 crosstalk in cancer

**DOI:** 10.18632/oncotarget.15367

**Published:** 2017-02-16

**Authors:** José Yélamos

**Affiliations:** Cancer Research Program, Hospital del Mar Medical Research Institute, Barcelona, Spain

**Keywords:** DNA damage response, ATM, MAPK7

Cells have evoked mechanisms, globally named the DNA damage response (DDR), to detect, signals and repair DNA lesions, which are tightly coordinated with apoptotic or cell cycle arrest responses [1]. Defects in these mechanisms lead to genomic instability, a hallmark of cancer cells. While key components of DDR have now been identified, further research is required to determine how these components are regulated in a temporal, spatial, and DNA lesion-specific manner. Arguably, most of our knowledge about DDR is centered on phosphorylation-based signaling pathways, which begin with the activation of the so-called PI3-kinase related kinases (PIKK). In particular, three PIKKs have been found to be involved in genome maintenance: Ataxia-Telangiectasia Mutated (ATM), ATM and Rad3-related (ATR) and the DNA-dependent protein kinase catalytic subunit (DNA-PKcs) [2]. Whereas the role of DNA-PKcs seems to be restricted to stimulating DNA repair through Non-Homologous End Joining (NHEJ), ATM and ATR additionally participate in the checkpoint response, namely, by restraining the expansion of damaged cells by stopping cell cycle progression. To coordinate such a large variety of activities, ATM and ATR phosphorylate hundreds of proteins, including the checkpoint kinases CHK2 and CHK1, respectively, which are key mediator of the checkpoint function [2]. Besides phosphorylation, other post-translational modifications such as methylation, acetylation, and PARylation have emerged as critical regulators of DDR [3].

Consistent with ATM playing a key role in DDR, ATM-null (*Atm*^−/−^) mice develop spontaneous thymic lymphomas. Likewise, ATM germline mutations predispose to cancer in humans, while somatic ATM mutations are the most common abnormalities found in chronic lymphocytic leukemia patients and are also present in about 5% of solid tumours [4]. A major challenge is now to determine how ATM interacts with other DDR components to modulate DNA repair, cell cycle checkpoint activation, and apoptosis. Being able to deregulate DDR by taking advantage of synthetic lethality holds enormous promise for novel cancer treatments [5]. Synthetic lethality refers to cell death that is caused by the simultaneous presence of two or more defects in different genes or pathways but not by each defect individually [6]. In of the best-known examples, this strategy was successfully used to take advantage of the synthetic lethal effects of PARP inhibitors on BRCA1/2-deficient breast and ovarian tumors. Interestingly, mice harboring a double deficiency for ATM and PARP-1, PARP-2, or H2AX are embryonic lethal [4], while p53 deficiency accelerates tumorigenesis in *Atm*^−/−^ mice [4].

Using mouse models, Granados-Jaén et al. [7] have now uncovered a functional interaction during tumorigenesis between ATM and MAPK7, a mammalian mitogen-activated protein kinase (also termed ERK5). MAPK7 has been shown to play a role in survival and growth of tumor cells. It has been implicated in diverse types of cancers, suggesting it could be promising novel target [8]. However, its functional interactions with DDR components are largely unknown. Loss of MAPK7 in *Atm*^−/−^ mice restores the DNA damage-signaling pathway in thymocytes, leading to H2AX phosphorylation, G2/M arrest, and increased apoptosis of thymocytes. The final result is a reduction of DNA instability that probably contributes to the delay of thymic lymphoma in the double-knockout mice [7]. Questions remain regarding tissue specificity of the ATM and MAPK7 functional interaction, as both kinases contribute to B cell development, but only MAPK7 is necessary for normal erythroid development in young mice [7].

In the meantime, the discoveries of Granados-Jaén et al. [7] also offer new potential therapeutic approaches, with the possibility of using MAPK7 inhibitors in cancer therapy to re-establish cell cycle checkpoints either in combination with ATM inhibitors or in ATM-deficient lymphomas. Certainly, a better understanding of DDR component network interactions must be attained to provide a basis for the development and rational exploitation of novel therapeutic strategies to fight cancer.
